# Influence of integrated molecular pathology test results on real-world management decisions for patients with pancreatic cysts: analysis of data from a national registry cohort

**DOI:** 10.1186/s13000-016-0462-x

**Published:** 2016-01-20

**Authors:** David Loren, Thomas Kowalski, Ali Siddiqui, Sara Jackson, Nicole Toney, Nidhi Malhotra, Nadim Haddad

**Affiliations:** Department of Medicine, Jefferson Digestive Disease Institute, Thomas Jefferson University, Philadelphia, PA USA; Interpace Diagnostics Corporation, 2515 Liberty Avenue, Pittsburgh, PA 15222 USA; Division of Gastroenterology, MedStar Georgetown University Hospital, Washington, DC, USA

**Keywords:** Endoscopic-ultrasound guided fine-needle aspiration, Integrated molecular pathology, Pancreatic cyst, Pancreatic neoplasm, Patient registry, Real-world management decisions, Sendai 2012 International consensus guideline

## Abstract

**Background:**

Integrated molecular pathology (IMP) approaches based on DNA mutational profiling accurately determine pancreatic cyst malignancy risk in patients lacking definitive diagnoses following endoscopic ultrasound imaging with fine-needle aspiration of fluid for cytology. In such cases, IMP ‘low-risk’ and ‘high-risk’ diagnoses reliably predict benign and malignant disease, respectively, and provide improved risk stratification for malignancy than a model of the 2012 International Consensus Guideline (ICG) recommendations. Our objective was to determine if initial adjunctive IMP testing influenced future real-world pancreatic cyst management decisions for intervention or surveillance relative to ICG recommendations, and if this benefitted patient outcomes.

**Methods:**

Analysis of data from the previously described National Pancreatic Cyst Registry. Associations between real-world decisions (intervention vs. surveillance), ICG model recommendations (surgery vs. surveillance) and IMP diagnoses (high-risk vs. low-risk) were evaluated using 2 × 2 tables. Kaplan Meier and hazard ratio analyses were used to assess time to malignancy. Odds ratios (OR) for surgery decision were determined using logistic regression.

**Results:**

Of 491 patients, 206 received clinical intervention at follow-up (183 surgery, 4 chemotherapy, 19 presumed by malignant cytology). Overall, 13 % (66/491) of patients had a malignant outcome and 87 % (425/491) had a benign outcome at 2.9 years’ follow-up. When ICG and IMP were concordant for surveillance/surgery recommendations, 83 % and 88 % actually underwent surveillance or surgery, respectively. However, when discordant, IMP diagnoses were predictive of real-world decisions, with 88 % of patients having an intervention when ICG recommended surveillance but IMP indicated high risk, and 55 % undergoing surveillance when ICG recommended surgery but IMP indicated low risk. These IMP-associated management decisions benefitted patient outcomes in these subgroups, as 57 % had malignant and 99 % had benign outcomes at a median 2.9 years’ follow-up. IMP was also more predictive of real-world decisions than ICG by multivariate analysis: OR 11.4 (95 % CI 6.0 − 23.7) versus 3.7 (2.4 − 5.8), respectively.

**Conclusions:**

DNA-based IMP diagnoses were predictive of real-world management decisions. Importantly, when ICG and IMP were discordant, IMP influence benefitted patients by increasing confidence in surveillance and surgery decisions and reducing the number of unnecessary surgeries in patients with benign disease.

## Background

Although pancreatic cystic lesions carry an increased overall risk for malignancy, the majority have a benign course, with an estimated malignant transformation rate of 0.4 % per year in cysts that are non-malignant at diagnosis [1, 2]. International and European groups have published consensus recommendations for pancreatic cyst management based on first-line test results (cytology, imaging, fluid chemistry) [3, 4], although recommendations are based mainly on specific cyst histology, which is frequently indiscernible without surgery [5]. An evidence-based approach was recently proposed by the American Gastroenterological Association, although the authors noted that the available evidence was of very low quality [5, 6]. Cytological analysis of samples collected by endoscopic ultrasound with fine-needle aspiration (EUS-FNA) has a high true positive rate for presence of malignancy (specificity 90–100 %) but a low true negative rate (sensitivity < ~40 %), and is often indeterminate [3, 7–10]. Given the poor survival rate for pancreatic carcinoma [11], management is often cautious, with many patients undergoing surgery, although only a minority have malignant cysts on surgical pathology [12–16]. A recent estimate derived from a pooled analysis of published literature indicated an invasive adenocarcinoma frequency of 15 % (95 % confidence interval [CI], 12–18 %) in patients with any type of pancreatic cyst undergoing surgery [5]. Thus, there is a need for a diagnostic approach that can reliably distinguish cysts that are malignant or likely to become malignant from those that will remain benign, in order to better ascertain which patients require surgery.

The widely-used Sendai 2012 International Consensus Guideline (ICG) recommendations [3] are based on traditional imaging, cytology and fluid chemistry criteria. Studies based on actual patient outcomes show that the ICG criteria for surgery versus surveillance have high sensitivity for malignancy but a high false positive rate; thus a substantial number of patients undergo unnecessary surgery [17–19]. Recent estimates of mortality and morbidity associated with pancreatic cyst resection based on a pooled literature analysis indicate that surgery is not without harm, even when conducted in specialized centers; the overall morbidity rate was 30 % (95 % CI, 25–35 %) and the mortality rate of 2.1 % (95 % CI, 1.5–2.7 %) was noted as a likely underestimation [5].

DNA mutational analysis of cyst fluid has identified features that correlate with malignancy, although they do not reliably predict malignancy risk when assessed individually [20]. Integrated molecular pathology (IMP) interrogates a panel of mutational changes in DNA (loss of heterozygosity mutations at 10 genomic loci, oncogene point mutation, DNA quantity) in the context of traditional first-line test results (cytology, fluid chemistry, imaging) to determine risk of malignancy [17, 21], and is intended to be used as an adjunct to first-line testing in patients who are indeterminate for malignancy following EUS imaging and FNA of pancreatic cyst/duct fluid for cytology. The performance of IMP in determining malignancy risk was assessed in the National Pancreatic Cyst Registry study, which reviewed follow-up medical records of patients who had an IMP test as a component of their real-world care, and showed that the IMP diagnostic categories of ‘benign (BEN)’ and ‘statistically indolent (SI)’ reliably predicted benign outcomes and ‘statistically higher risk (SHR)’ and ‘aggressive (AGG)’ reliably predicted malignant outcomes [17]. Compared with a model of the ICG criteria, IMP diagnoses increased the accuracy of surgery versus surveillance recommendations. IMP correctly recommended surveillance in 84 % of patients with benign outcomes for whom surgery was recommended based on ICG criteria, and also confirmed that ICG recommendations for surveillance were appropriate in 98 % of patients (benign outcomes) [17]. IMP recommended surgery in four of six patients with malignancies that were missed by ICG criteria (i.e. lacking ‘worrisome’ features) [17]. The use of IMP testing as an adjunct to guideline-recommended criteria may help limit false negatives and increase confidence that surveillance at longer intervals is appropriate in most patients [17, 22].

Because IMP is associated with a per-patient cost that is greater than that of first-line tests alone, the economic utility of IMP has been assessed in order to determine its overall cost effectiveness. Using a Markov decision model with a third-party-payer perspective to compare management strategies, which included resecting only mucinous cysts based on guideline-recommended first-line testing alone or resecting only cysts with an AGG IMP diagnosis, Das et al. found that an IMP-guided management strategy was the most cost-effective approach that provided the greatest increase in quality-adjusted life years to patients by limiting the number of surgeries on patients with benign disease course while accurately diagnosing malignant disease [23].

The objective of this analysis was to determine if the adjunctive use of DNA-based IMP testing in the initial clinical diagnosis of cystic lesions influenced real-world pancreatic cyst management decisions at follow-up relative to the initial ICG recommendations alone, and if so, whether the changes were of benefit to patients with respect to their outcome (i.e. reduction in unnecessary surgery). We analyzed data from the National Pancreatic Cyst Registry cohort to determine the association between real-world physician decision for clinical intervention (i.e. surgery or chemotherapy), or surveillance and recommendations based on initial IMP diagnoses or a model of the traditional ICG criteria.

## Methods

### Study design and patient population

This was an analysis of data from the previously described National Pancreatic Cyst Registry, which included adults who underwent EUS-FNA of a pancreatic cyst and had negative, non-diagnostic, indeterminate or acellular EUS-FNA cytology results. In these patients, IMP was performed as a part of clinical testing per the prescribing physician’s standard of care [17]. After initial IMP testing, all available clinical data pertaining to the pancreatic lesion were abstracted from medical records into a database in a standardized manner, without additional interpretation. Ethical approval was obtained at each site as previously described with approval for continued data analysis described here maintained by the central IRB (Quorum Review IRB) [17].

IMP diagnosis (PancraGen™ using PathFinder®, Interpace Diagnostics Corporation, Pittsburgh, PA, USA) was made according to standard operating procedures at Interpace prior to inclusion of patient information into the registry, and thus without knowledge of the patient’s actual clinical outcome. Molecular analysis was performed as described previously [17, 20, 21]. In brief, quantitative molecular pathology parameters included DNA quantity/quality, presence of oncogene (*KRAS*) point mutation and extent of clonal expansion, and presence of tumor suppressor gene loss of heterozygosity (LOH) and extent of clonal expansion, as determined by allelic imbalance. The following chromosomal loci were examined for LOH (associated genes in parentheses): 1p (*CMM1*, *L*-*myc*), 3p (*VHL*, *HoGG1*), 5q (*MCC*, *APC*), 9p (*CDKN2A*), 10q (*PTEN*, *MXI1*), 17p (*TP53*), 17q (*NME1*), 18q (*DCC*), 21q (*TFF1* and *PSEN2*), and 22q (*NF2*). The method for determining the presence and clonal expansion of oncogene point mutations and LOH mutations has been described previously [20, 24]. IMP integration of first-line test results (cytology, fluid chemistry, imaging) with these molecular parameters has been described elsewhere [17].

Patient outcomes at follow-up were determined as described previously [17]. Benign outcomes included benign or low/intermediate-grade dysplasia on surgical pathology, cyst resolution on repeat imaging, or follow-up of more than 23 months without evidence of malignancy. Malignant outcomes were malignant cytology results (unknown during IMP diagnosis), high-grade dysplasia (HGD), or clinically confirmed pancreatic cancer.

### Definitions of clinical intervention and surveillance decisions

Real-world decisions were defined as clinical ‘intervention’ if any of the following outcomes occurred within 12 months of the index EUS-FNA: surgical report and/or surgical pathology, chemotherapy, or positive cytology. If none of the above ‘intervention’ conditions were met, the real-world decision was determined to be ‘surveillance’. For IMP, diagnoses of SHR and AGG were categorized as ‘high-risk’, with BEN and SI being categorized as ‘low-risk’. The ICG criteria model has been described in detail previously [17]: if there were no high-risk stigmata or worrisome features then cases were classed as a ‘surveillance recommendation’; all other cases were classed a ‘surgery recommendation’.

### Statistical analysis

Associations of IMP diagnoses (high-risk vs. low-risk) and ICG recommendations (surgery vs. surveillance) with real-world decisions (intervention vs. surveillance) and actual patient outcomes (benign vs. malignant) were evaluated using 2 × 2 tables. Univariate and multivariate logistic regression models were used to determine the odds ratio (OR) for surgery decision and for malignancy; the 95 % CI was estimated using the profile-likelihood method. Kaplan − Meier analysis was used to determine time to malignant event, with probability of benign outcome after IMP test shown, and the hazard ratio (HR) for risk of malignancy calculated using a multivariate Cox proportional hazards model.

## Results and Discussion

This cohort comprised 492 patients who had cystic lesions indeterminate for malignancy, for whom further information has been reported previously [17]. Overall, 491 patients were included in this analysis; one patient whose outcome was death due to pancreatic cancer was excluded from the analysis due to lack of clear evidence for surveillance or surgical intervention at follow-up. Overall 19 % (66/491) of patients had malignant outcome and 87 % (425/491) of patients had benign outcome at follow-up. Median follow-up time for patients who underwent surveillance post initial EUS-FNA (*n* = 285) was 2.9 years (range, 1.9 − 7.7 years).

In total, 42 % (206/491) of patients received a clinical intervention less than 1 year after initial EUS-FNA (Table 1). Although we refer here to real-world ‘intervention’ decisions, the majority of these were surgery decisions, as 183 patients (89 %) had definitive reports of surgery in their records. Of the remaining cases, four (2 %) received chemotherapy and 19 (9 %) had presumed intervention (e.g. surgery or chemotherapy) due to frankly malignant cytology results recorded at follow-up. Thus, up to 98 % of patients in this cohort had surgery, depending on the proportion of patients with malignant cytology undergoing surgery. It seems logical to suppose that surgery would be the goal in all of these ‘intervention’ cases but may not proceed in patients who are unfit for or decline surgery, and thus receive chemotherapy instead.

**Table 1:** Comparison of real-world decisions with ICG recommendations and IMP diagnoses

*N* = 491	Intervention, %	Surveillance, %
Real-world decision at follow-up	42	58
Initial ICG recommendation	59	41
Initial IMP recommendation based on high or low risk diagnosis	19	81

A comparison of real-world decisions with IMP and ICG model recommendations is shown in Table 1. The real-world decisions at follow-up were approximately half-way between the initial ICG recommendations and IMP diagnoses, with IMP recommending the lowest proportion of patients for intervention.

Ideally, there should be a close correlation between cases with malignant outcomes at follow-up and cases recommended for intervention because they are initially deemed high risk by diagnostic testing. Similarly, there should be a close correlation between cases with benign outcomes and cases recommended for surveillance because they are initially deemed low risk by diagnostic testing. The actual proportions of patients in this cohort with malignant and benign outcomes were 13 % (66/491) and 87 % (425/491), respectively, which closely reflect those with initial IMP high-risk diagnoses who were recommended for intervention (19 %) and those with initial low-risk IMP diagnoses who were recommended for surveillance (81 %), respectively (Table 1).

**Fig. 1:**
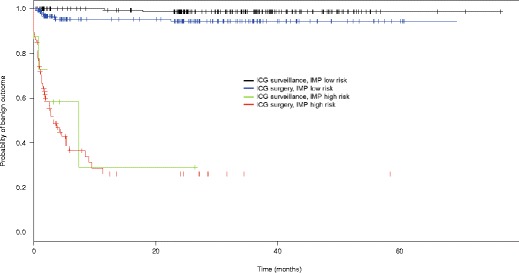
Kaplan-Meier analysis of time-to-malignancy in a multivariate model as predicted by IMP and ICG. Y-axis represents probability of benign outcome in patients after initial IMP test. X-axis represents time post initial EUS-FNA that patient outcomes were determined based on review of follow-up medical records

When surveillance was indicated by both ICG recommendations and IMP low-risk diagnoses, 83 % of patients actually underwent surveillance, which was the appropriate decision in the large majority as 99 % of this group (161/162) had benign outcomes (Table 2). Kaplan − Meier analysis confirmed high probability of benign disease at follow-up in these patients (Fig. 1; black line). When intervention was indicated by both ICG and IMP, 88 % of patients actually underwent intervention within 1 year of IMP testing, with the majority of patients benefiting as 66 % (50/76) had malignant outcomes (Table 2). Kaplan − Meier analysis confirmed low probability of benign disease at follow-up in these patients (Fig. 1; red line).

**Table 2:** IMP diagnoses influenced real-world intervention and surveillance decisions that were of benefit to patients (total *N* = 491)

Initial ICG recommendation	Initial IMP diagnosis	Patients who had clinical intervention at follow-up in reality			Patients who had surveillance at follow-up in reality^a^		
		n	Surgery rate, %	Malignant outcome rate, %	n	Surveillance rate, %	Benign outcome rate, %
Surveillance	Low risk (*n* = 195)	33	17	3	162	83	99
	High risk (*n* = 8)	7	88	57	1	12	100
Surgery	Low risk (*n* = 202)	90	45	9	112	55	99
	High risk (*n* = 86)	76	88	66	10	12	100

^a^Median follow-up time 2.9 years (range, 1.9 to 7.7 years)

However, when ICG diagnoses and IMP recommendations were discordant, real-world clinical intervention and surveillance decisions at follow-up were more reflective of initial IMP diagnoses than initial ICG criteria recommendations. Patients were more likely to undergo intervention when IMP diagnoses indicated high risk and more likely to undergo surveillance when IMP diagnoses indicated low risk. When the ICG recommendation was surveillance but IMP diagnosis indicated high risk, 88 % of patients actually had an intervention within 1 year of IMP testing, suggesting that IMP influenced the decision for intervention (Table 2). This decision was of benefit to the majority of these patients, as 57 % had malignant outcomes (Table 2). Kaplan − Meier analysis confirmed that IMP high-risk diagnoses reliably predicted the need for intervention even when ICG criteria recommended surveillance: conditional HR for malignancy = 29.5; *p* < 0.0001 (Fig. 1; green vs. black line). IMP can therefore provide evidence for intervention need in cases where ICG criteria falsely indicate that surveillance is appropriate. Furthermore, when ICG recommended surgery but IMP diagnosis indicated low risk, 55 % of patients underwent surveillance in real-life, suggesting that IMP facilitated more liberal surveillance decisions, which was of benefit to the vast majority of these patients as 99 % had benign outcomes at a median of ~3 years’ follow-up (Table 2). ICG did not predict the need for intervention in cases where IMP indicated low risk; conditional HR for malignancy = 2.3; *p* = 0.08 (Fig. 1; blue vs. black line).

When applied to management decisions for pancreatic cysts, IMP increases the confidence in surveillance decisions where ICG criteria frequently incorrectly indicate a need for intervention. A reduction in the number of unnecessary surgeries is a key aim of improving pancreatic cyst management, given the surgical morbidity risk, and particularly the mortality risk of ~2 % at even the most specialized academic institutions [5]. Thus, the information provided by the adjunctive use of IMP allows physicians to make better-informed decisions that ultimately benefit the health of their patients by more accurately balancing the mortality of pancreatic cancer with the risks of surgical resection.

While it is clear that adjuvant IMP diagnoses can provide a more complete picture of risk for malignancy than guideline criteria alone, and that the test can influence follow-up surveillance and intervention decisions for patients, the real-world intervention rate was higher than that recommended by IMP in this cohort, although it remained lower than that recommended by ICG (Table 1). Physician clinical judgment and patient preferences play a critical role in decision making, and these factors could account for the discrepancy between the objective data derived from IMP or ICG and the decision to proceed to surgery. Additional clinical circumstances of the patient (e.g. suspicious clinical history or comorbidities, or presence of known risk factors for pancreatic cancer) are likely to influence this judgment and preference, as patients with pancreatic cysts in the real world are managed on a case-by-case basis.

Univariate analysis confirmed that initial IMP diagnoses were more predictive of actual decisions for intervention and surveillance than ICG recommendations (OR = 16.8 [95 % CI 9.0 − 34.4] for IMP vs. 5.6 [3.7 − 8.5] for ICG), and this remained so after adjusting for ICG criteria in a multivariate analysis (Table 3), showing that initial IMP diagnoses were significantly predictive of real-world decisions at follow-up. IMP diagnoses were also highly predictive of actual benign and malignant patient outcomes by univariate analysis, more than traditional clinical criteria (OR = 47.4 for IMP vs. 8.5 for ICG; both *p* < 0.0001), an association that remained highly significant for IMP after controlling for ICG recommendations in a multivariate analysis (adjusted OR = 35.8, *p* < 0.0001 for IMP vs. 2.5, *p* = 0.066 for ICG).

**Table 3:** IMP diagnoses were highly associated with real-world decisions

Association	Univariate analysis		Multivariate analysis	
	OR (95 % CI)	*p* value	OR (95 % CI)	*p* value
*Real*-*world decision* (*intervention* vs. *surveillance*) *at follow*-*up*				
Initial ICG recommendation	5.6 (3.7 − 8.5)	<0.0001	3.7 (2.4 − 5.8)	<0.0001
Initial IMP diagnosis	16.8 (9.0 − 34.4)	<0.0001	11.4 (6.0 − 23.7)	<0.0001
*Actual patient outcome* (*benign* vs. *malignant*) *at follow*-*up*				
Initial ICG recommendation	8.5 (3.9 − 22.3)	<0.0001	2.5 (1.0 − 7.4)	0.066
Initial IMP diagnosis	47.4 (23.8 − 102.4)	<0.0001	35.8 (17.4 − 80.0)	<0.0001

Univariate and multivariate analyses indicated that IMP diagnoses were highly associated with real-world intervention and surveillance decisions and predictive of actual patient outcomes

We also performed an analysis to determine if the molecular criteria that are unique to the IMP test (*KRAS* mutation, DNA quantity, loss of heterozygosity mutations) [17] were associated with real-world decisions for intervention and surveillance. There was a clear trend towards an association between real-world decisions for intervention in patients and high levels of each of these molecular parameters. Univariate logistic regression indicated that a combination of any 2 of these high risk molecular criteria incorporated into an IMP high-risk diagnosis was associated with real-world decisions for intervention in patients (*p* = 0.048). These findings suggest that it is this molecular component of IMP that produces the strong association between future real-world decisions and initial IMP diagnoses.

As discussed elsewhere, a limitation of this analysis is that the outcomes data were obtained via review of medical records from patients previously tested by IMP [17]. However, due to the benign nature of the majority of pancreatic cysts, this approach is currently the only practical method of conducting studies to assess pancreatic cyst outcomes in clinical practice. There are some minor differences between our ICG model and the published criteria, reflecting the patient cohort (those who have undergone EUS-FNA and have been prescribed IMP testing for indeterminate cystic lesions) and the study design (real-world medical record review); however, accounting for these differences were shown to have minimal impact on the performance of the ICG model in our study and no impact on the overall conclusions of the study [25].

## Conclusion

In this cohort of patients with pancreatic cystic lesions indeterminate for malignancy by EUS-FNA, real-world intervention and surveillance decisions were highly associated with the initial DNA-based IMP diagnostic recommendations. Initial IMP diagnoses were more predictive of future, real-world patient management decisions and more beneficial to overall patient outcomes than solely relying on traditional EUS-FNA criteria recommended by ICG. When used as an adjuvant to traditional EUS-FNA testing, IMP increases physician confidence in the decision to proceed with surgery and reduces the number of unnecessary surgeries performed for benign disease.

## Abbreviations

AGG: IMP diagnosis of ‘aggressive’
BEN: IMP diagnosis of ‘benign’
CI: confidence interval
EUS-FNA: endoscopic ultrasound with fine-needle aspiration
HGD: high-grade dysplasia
HR: hazard ratio
ICG: Sendai 2012 International Consensus Guideline
IMP: integrated molecular pathology
OR: odds ratio
SHR: IMP diagnosis of ‘statistically higher risk’
SI: IMP diagnosis of ‘statistically indolent’
